# Quality Assessment of Colonoscopy Reporting: Results from a Statewide Cancer Screening Program

**DOI:** 10.1155/2010/419796

**Published:** 2010-09-28

**Authors:** Jun Li, Marion R. Nadel, Carolyn F. Poppell, Diane M. Dwyer, David A. Lieberman, Eileen K. Steinberger

**Affiliations:** ^1^Division of Cancer Prevention and Control, Centers for Disease Control and Prevention, 4770 Buford Hwy, Mail Stop K-55, Atlanta, GA 30341, USA; ^2^Department of Epidemiology and Preventive Medicine, University of Maryland, 21201 Baltimore, MD, USA; ^3^Department of Health and Mental Hygiene, 21201 Baltimore, MD, USA; ^4^Division of Gastroenterology and Hepatology, Department of Medicine, Oregon Health and Science University, 97239 Portland, OR, USA

## Abstract

This paper aimed to assess quality of colonoscopy reports and determine if physicians in practice were already documenting recommended quality indicators, prior to the publication of a standardized Colonoscopy Reporting and Data System (CO-RADS) in 2007. We examined 110 colonoscopy reports from 2005-2006 through Maryland Colorectal Cancer Screening Program. We evaluated 25 key data elements recommended by CO-RADS, including procedure indications, risk/comorbidity assessments, procedure technical descriptions, colonoscopy findings, specimen retrieval/pathology. Among 110 reports, 73% documented the bowel preparation quality and 82% documented specific cecal landmarks. For the 177 individual polyps identified, information on size and morphology was documented for 87% and 53%, respectively. Colonoscopy reporting varied considerately in the pre-CO-RADS period. The absence of key data elements may impact the ability to make recommendations for recall intervals. This paper provides baseline data to assess if CO-RADS has an impact on reporting and how best to improve the quality of reporting.

## 1. Introduction

Colorectal cancer (CRC) screening reduces CRC incidence and mortality [1–6]. Major national organizations such as the United States Preventive Services Task Force (USPSTF), the American Cancer Society, and the Multi-Society Task Force on Colorectal Cancer (MSTF-CRC) recommend that average-risk adults begin CRC screening at age 50 [7, 8]. Any positive CRC screening test leads to a colonoscopy, which itself can be a primary CRC screening tool [7, 8]. In the last few years, utilization of colonoscopy for CRC screening has increased dramatically [9–11].

The effectiveness of colonoscopy in reducing CRC incidence and mortality depends on many factors. These include adequate visualization of the entire colon, diligence in examining the mucosa, and patient acceptance of the procedure [12]. Many clinical practice studies have found variations in colonoscopy quality [13–15] which has become a concern in the gastroenterology community. In 2002, the MSTF-CRC published recommendations to improve the quality and effectiveness of colonoscopy and highlighted key indicators for continuous quality improvement (CQI) [16]. In order to measure indicators of quality, guidelines for reporting the indicators need to be in place. 

Colonoscopy reporting practices are widely variable [17]. Appropriate documentation of the colonoscopy procedure is an important component of patient care and a key approach to measure health care quality [17, 18]. Measurement of quality indicators for colonoscopy reporting can identify areas for quality improvement [19]. To facilitate such quality measurement within and across practices, the Quality Assurance Task Group of the National Colorectal Cancer Roundtable (NCCRT) developed, and in 2007 published, a standardized Colonoscopy Reporting and Data System (CO-RADS) [20]. CO-RADS describes the specific elements for inclusion in all colonoscopy reports, recommends standard terms for procedure reporting, and suggests key quality indicators. Standardized language also facilitates communication between the endoscopist and the primary care provider, who is often responsible for referring the patient for the next surveillance or screening colonoscopy. Similar standardized reporting and data systems are in place for both Pap tests (the Bethesda System) and mammography [21, 22]. 

To our knowledge, only a few studies have directly evaluated colonoscopy reporting [17, 19, 23]. Most recently, Lieberman et al. examined colonoscopy reports from the 73 Clinical Outcomes Research Initiative gastroenterology practice sites in the United States that used a structured, computerized endoscopy report generator [19]. The aim of this paper was to assess the quality of colonoscopy reports and determine if endoscopists in practice were already documenting recommended quality indicators, prior to the publication of CO-RADS. This paper may provide baseline data to assess if CO-RADS has an impact on reporting and how best to improve the quality of reporting.

## 2. Materials and Methods

We selected colonoscopy reports for review from a database containing information related to colonoscopies performed through Maryland's Cigarette Restitution Fund Colorectal Cancer Screening Program. Under this program, in 2005 and 2006, 22 of 24 local health departments (LHDs) offered CRC screening targeted to Maryland residents who were 50–64 years old, with low income, and uninsured. Eighty-one percent (81%) of the targeted population in Maryland resided in these 22 jurisdictions. Most of those jurisdictions accepted patients with lower-gastrointestinal symptoms such as abdominal pain, constipation, and blood in stool. LHDs receive the provider's routine colonoscopy reports and enter results into a secure intranet-based database maintained at the Maryland Department of Health and Mental Hygiene (MDHMH) for local case management, program statistics, and quality assurance. Institutional Review Boards at the MDHMH and the University of Maryland exempted this study. 

Reports eligible for review were from colonoscopies that were (1) performed from Jan 1, 2005 through Dec 31, 2006, (2) performed on patients having their first colonoscopy in this program, and (3) had findings of polyp(s) so that we could measure as many of the quality indicators as possible per report. There were 788 eligible colonoscopies with a finding of one or more polyps (adenomatous, hyperplastic, or other type of polyp) during 2005-2006 performed by 110 different endoscopists (gastroenterologists or surgeons) from community endoscopy practices: 38 endoscopists performed 1 or 2 procedures that met these criteria, and 72 performed 3 or more procedures. One eligible colonoscopy was sampled from each of the 110 endoscopists. If an endoscopist is named on only one eligible colonoscopy report, that report is reviewed. For those in the database who performed two or more colonoscopies on program patients, one report was randomly selected. For each selected colonoscopy, staff at the MDHMH requested a copy of the colonoscopy report from the patient medical record maintained at the LHDs. Before the reports were sent to the MDHMH for analysis, the local program staff removed all identifying information about the patient, doctor, and facility. No patient or physician information could be associated with the reports. 

We evaluated 25 of the key data elements recommended by CO-RADS, including procedure indications, risk and comorbidity assessments, procedure technical description, colonoscopy findings (including polyp characteristics), specimen retrieval, and submission for pathologic examination. In some reports, the status of specimen retrieval and submission for pathology were not directly documented, but were inferred from phrases such as “wait for histopathology,” “follow up on pathology,” and “follow up biopsy results”. We created a standardized data form using Microsoft Access to abstract information on each element. Two investigators, one from CDC and one from the MDHMH, reviewed the reports and extracted the data elements independently. Any differences between the two abstractors arising during the extraction process were resolved by consensus of these two investigators. To determine counts and percentages, we exported the final database into Microsoft Excel and SAS (version 9.1).

## 3. Results

The sample of 110 colonoscopy reports from 2005-2006 in which one or more polyps had been described represented 110 different endoscopists from community endoscopy practices throughout Maryland. We could not assess the documentation of patient's age and sex; in an effort to ensure data anonymity, LHD staff had removed this information from some reports. 

As shown in Table 1, all reports documented the indication for the procedure; 19% were done for screening where the patients were cited as being either average or increased risk; 41% were done for screening where no patient risk was cited; the remaining 40% were done either for reported symptoms, family history, as surveillance colonoscopies, or as followup to an abnormal screening test. The patient's medical comorbidities were mentioned in 36% (*n* = 40) of the reports, and 15% (*n* = 16) used American Society of Anesthesiologists (ASA) classification. As shown in Table 2, the quality of the bowel preparation was noted in 73% (*n* = 80) of reports. The quality was described as “excellent,” “good,” “well prepared”, “poor”, or “inadequate” in 59 reports. Other terms used to describe the bowel preparation included “fair", “adequate”, or “suboptimal." Ten reports documented the type of preparation used. About 71% of the reports (*n* = 78) had specific sedation medication names; 71 of these included the dose given. The health provider who administered the sedation was documented in 27 reports, and anesthesiologists or nurse anesthetists administered the sedation in 22 of these reports. Only two colonoscopy reports included no information related to sedation. 

As shown in Table 3, 108 (98%) reports documented the extent or completeness of the examination. Seventy-nine (72%) reports documented at least one cecal landmark using the terms “ileocecal valve,” “appendiceal orifice,” and/or “terminal ileum.” Other terms used solely to describe the intubation of the cecum included “light in right lower quadrant,” “landmark,” “anatomic configuration,” or “caput.” Only half or fewer reports included information about photodocumentation of cecal landmarks, ease of performing the examination, and retroflexion of the endoscope on withdrawal from the rectum. Three reports documented total procedure time; one of these documented withdrawal time as well. 

There were 177 individual polyps documented among the 110 reports. Polyp location was documented for all but one of the individual polyps (Table 4): 82% (*n* = 145) were described by anatomic location within the colon, 8% (*n* = 15) were described by their distance in centimeters from the anal verge, and 9% (*n* = 16) were described by both methods. Of the 15 described by distance from the anal verge, three were located in the rectosigmoid segment. Information on polyp size was provided for 154 (87%) polyps: polyp size in millimeters or centimeters for 66 polyps, descriptive terms for 52, and by both methods for 36 polyps. Where both text and numeric descriptions were used, we found that different physicians did not use the descriptive terms in the same way. For example, some physicians reported a 2–5 mm polyp as “small,” while others described it as “diminutive.” Of four polyps measured as 10 mm, three were described as “small,” and the fourth described as “large.” Polyp morphology was documented for 94 (53%) individual polyps. Of these, the terms “pedunculated,” “sessile,” or “flat” were used to describe 73 of the polyps. Information on whether the polyp was biopsied or removed was documented for 174 (98%) polyps; the method of biopsy or removal was reported for 169 (95%) polyps. The method was described as “snare with cautery,” “cold biopsy,” “hot biopsy,” and “fulguration/ablation” for 90 polyps; the method was solely described as “snare,” “biopsy,” “forceps,” and “cautery” for the remaining 79 polyps. 

## 4. Discussion

In this study, we looked at 110 colonoscopy reports sampled from endoscopists in 22 of Maryland's 24 jurisdictions, for a time period of 3 to 4 years after publication of the MSTF-CRC recommendations but before the publication of CO-RADS. We found that certain key quality indicators were not consistently well documented by endoscopists in their colonoscopy reports prior to the publication of CO-RADS including patient risk and comorbidity, quality of the bowel preparation, and polyp size and morphology.

CO-RADS recommends use of the ASA classification to document risk and comorbidity [20]. For patients with an ASA class 3 or higher, colonoscopy should be performed in a hospital or a setting with full capacity for resuscitation and support [20]. In our study, almost two thirds of reports lacked any comorbidity documentation. Only 15% of reports provided the ASA class. While most patients were seen for a prescreening or complete physical exam prior to the endoscopy, where the endoscopist reviews the patient's prior history and documents it in the office record, this information was not always included in the colonoscopy report. 

It has been reported that interval cancers within 1 to 4 years of screening colonoscopy could result from missed lesions or incomplete adenoma removal [24]. In fact, computed tomographic colonography studies have shown that optical colonoscopy missed 2%–12% of polyps larger than 10 mm [25–27]. Missing lesions may be attributed to inadequate bowel preparation, an incomplete procedure, or failure to identify a lesion due to inadequate time spent examining the colonic mucosa. Inadequate bowel preparation not only limits the visibility of the mucosa and prolongs cecal intubation time and withdrawal time, but it also leads to a shorter interval for the next exam [12]. Our results revealed that about 25% of reports lacked any mention of bowel preparation quality and another 20% of reports used ambiguous terms to describe the quality. We have heard anecdotally from some programs that some endoscopists “chart by exception”, meaning they only mention the quality of the bowel preparation when it is inadequate. However, CO-RADS recommends that endoscopists explicitly document whether they believe bowel preparation was adequate to allow the detection of lesions larger than 5 mm [20]. 

Ensuring complete examination of the colon helps reduce the possibility of missing lesions. All colonoscopy reports in our study mentioned the extent of the examination; however, 18% did not provide information on specific cecal landmarks. Short withdrawal time may be associated with detecting fewer adenomas [28]. We found only one report that documented the withdrawal time. However, our study period predated the publication of the association between withdrawal time and adenoma detection rates and the subsequent recommendation to document the withdrawal time in colonoscopy reports. 

Polyp characteristics are important factors in assessing risk for malignancy and recurrence of advanced lesions, and in determining the follow-up interval [29, 30]. In this study, size information was absent for 13% of individual polyps. In up to one third of individual polyps, size was described solely with qualitative terms such as “small” or “large.” Moreover, different endoscopists used different qualitative terms to describe same-sized polyps. CO-RADS recommends reporting polyp size in millimeters. This facilitates determination of the appropriate interval for follow-up exams and clear communication between the endoscopist and the referring physician and patient. While exact measurement of polyp size during the endoscopy may be difficult—given that the endoscope contains no measuring device—many endoscopists use the open biopsy forceps to estimate polyp size. According to CO-RADS, polyp morphology should be documented as pedunculated, sessile, or flat. In our study these specific morphologic descriptions were absent for almost half of the identified individual polyps, though other descriptors were often used. It may be that some endoscopists only include morphologic descriptions for larger polyps where the morphology was clearly sessile, flat, or pedunculated. Indication of whether a polyp was completely removed is a key factor to predict the risk for recurrence of an adenoma and the risk for CRC, and to set the recall interval. CO-RADS recommends including this information in the colonoscopy report. However, this was not always clear in our study. Although “removed” probably meant “completely removed” in many places, we recommend that endoscopists assess the completeness of removal of each polyp (or lack thereof) and clearly state this in the colonoscopy report. 

To our knowledge, only a few studies have directly evaluated quality in colonoscopy reporting [17, 23]. In 2002, Robertson et al. assessed quality of colonoscopy reporting by reviewing a single colonoscopy report from 122 independent endoscopy centers across the nation [17]. Overall, the quality of colonoscopy reporting in Robertson's study was poorer than what we found in our study. In Robertson's study, all colonoscopy procedures were performed on colorectal cancer patients to detect cancer recurrence [17]. The study claimed that local customs and template sharing may result in less variation within and across healthcare facilities [17]. For our study, we used data from colonoscopies performed throughout a statewide colorectal cancer screening program. This program routinely collects available information on extent of examination, quality of bowel preparation, and number, size, and type of polyps. LHD may have emphasized the need for these data elements for program-patient management over the years. Recently, Lieberman et al. evaluated the quality of colonoscopy reports in 73 U.S. gastroenterology practice sites that used a structured computerized endoscopy report generator. In our study, we analyzed all types of reports, not just those from computerized report generators. Because of the standard use of a computerized report generator among the centers in the consortium, Lieberman's study was biased toward finding high rates of completion of quality indicators [19]. 

The findings of our study are subject to the following limitations. First, our study was not population based in that it did not include every endoscopist in Maryland; it did, however, include 110 gastroenterologists and surgeons from throughout Maryland who were contracted in the CRC screening program. Second, inclusion of only one report per endoscopist may not adequately represent his/her reporting quality or the change in quality over time. 

Many endoscopists use a reporting tool to generate their reports. The format and choices in such tools may not follow CO-RADS and may thus limit the ability of endoscopists to easily include all the CO-RADS elements. Also, the lower reporting rate of certain quality indicators on the colonoscopy report may reflect the fact that these items were routinely recorded in the other parts of the medical record and not duplicated in the colonoscopy report. The colonoscopy team includes the endoscopist, anesthetist or anesthesiologist, and nurses and technicians providing care to the patient. The patient's medical history is reviewed by the endoscopist and anesthesiologist and probably recorded elsewhere. If an anesthesiologist is administering the sedation, he or she will keep a separate record for the patient's chart. Nurses and technicians working in the endoscopy suite maintain records concerning the documentation of informed consent, type and upkeep of the endoscope, as well as specimens that are retrieved and sent to pathology. Nevertheless, the American Society for Gastrointestinal Endoscopy (ASGE) guideline published in 1999 suggested these elements should be included in a single colonoscopy report [17, 18]. 

## 5. Conclusions

This paper provides a snapshot of colonoscopy reporting in a wide variety of practices that use different reporting systems in pre-CO-RADS period. We observed considerable variation in reporting of key quality indicators. In some cases, the absence of key information may complicate a determination of the appropriate interval for follow-up exams. Intervals for surveillance which are longer than what might be recommended based on adequate information on polyp size, morphology, or completeness of removal may have a deleterious effect on colorectal cancer prevention. Intervals which are too short may expose the patient to unnecessary risk and cost. This paper also provides baseline data to assess if CO-RADS has an impact on colonoscopy reporting and how best to improve quality of reporting. Overall, these data highlight the importance of monitoring quality indicators in endoscopic practice, with the goal of improving colonoscopy quality. We encourage endoscopists to accept and implement the recommendations of CO-RADS to achieve this goal.

##  Disclosure

All authors stated that they had no financial relationships relevant to this publication. The findings and conclusions in this paper are those of the authors and do not necessarily represent the official position of the Centers for Disease Control and Prevention.

## Figures and Tables

**Table 1:** Counts and percentages of preprocedure data elements included in 110 colonoscopy reports.

		Count (*n*)	Percentage (%)
Informed consent	Included	75	68
	Not included	35	32

Indication for colonoscopy	Included	110	100
	Average or high risk screening	21	19
	Screening, no other indication	45	41
	Family history	9	8
	Surveillance	2	2
	Followup to a positive screening test	4	4
	Symptoms	29	26
	Not included	0	0

Risk and comorbidity	Included	40	36
	ASA classification	16	15
	“unremarkable physical exam", “stable cardiorespiratory system", specified diseases, or refer to other documents	24	21
	Not included	70	64

Abbreviations: ASA: American Society of Anesthesiology; RLQ: right lower quadrant.

**Table 2:** Counts and percentages of data elements for procedural preparations included in the 110 colonoscopy reports.

		Count (*n*)	Percentage (%)
Quality of bowel preparation	Included	80	73
	“Excellent," “good" or “well prepared"	52	47
	“Poor" or “inadequate"	7	6
	“Adequate"	7	6
	“Fair" or “suboptimal"	9	8
	“Regular," “stool" or “left side good"	5	5
	Not included	30	27

Bowel preparation type	Included	10	9
	Not included	100	91

Sedation medication name	Included	78	71
	Not included	32	29

Sedation medication dose	Included	71	65
	Not included	39	35

Sedation level	Included	17	15
	Not included	93	85

Sedation provider	Included	27	25
	Not included	83	75

Instrument type	Included	49	45
	Not included	61	55

Instrument number	Included	33	30
	Not included	77	70

**Table 3:** Counts and percentages of data elements for procedure findings included in 110 colonoscopy reports.

		Count (*n*)	Percentage (%)
Extent of colonoscopic exam	Included	108	98
	Cecum reached	107	97
	Cecum not reached	1	1
	Not included	2	2

Documentation of cecal landmarks	Included	90	82
	“Ileocecal valve," “appendiceal orifice" or “terminal ileum"	79	72
	“Light in RLQ," “landmark," “anatomic configuration" or “caput"	11	10
	Not included	20	18

Photodocumentation of landmarks	Included	32	29
	Not included	78	71

Ease of examination	Included	58	53
	Not included	52	47

Retroflexion	Included	51	46
	Not included	59	54

Total time	Included	3	3
	Not included	107	97

Withdrawal time	Included	1	1
	Not included	109	99

**Table 4:** Counts and percentages of data elements described for 177 individual polyps.

		Count (*n*)	Percentage (%)
Location	Included	176	99
	By distance only	15	8
	By segment only	145	82
	By both	16	9
	Not included	1	1

Size	Included	154	87
	By number (mm/cm) only	66	37
	By descriptive term only	52	29
	By both	36	20
	Not included	23	13

Morphology	Included	94	53
	“Pedunculated," “sessile", or “flat"	73	41
	“Benign," “benign and smooth", or “nonbleeding"	9	5
	“Adenoma-like," “hyperplastic," “villous-like," “polypoid," “firm mass," “Oblong," “more raised," “submucosal", or “irregular"	12	7
	Not included	83	47

Biopsy/removal	Included	174	98
	Not included	3	2

Methods	Included	169	95
	“Snare w/cautery," “cold biopsy," “hot biopsy," “fulguration" or “ablation"	90	51
	“Snare," “biopsy," “forceps" or “cautery"	79	45
	Not included	8	5

Retrieval^a^	Included	140^b^	80
	Not included	35	20

Pathology^a^	Included	134^C^	77
	Not included	41	23

^a^175 polyps were used for percentage calculation because 2 polyps were completely fulgurated.

^b^Information on specimen retrieval was inferred for 104 of the 140 individual polyps.

^C^Information on specimen submission for pathology was inferred for 95 of the 134 individual polyps.
